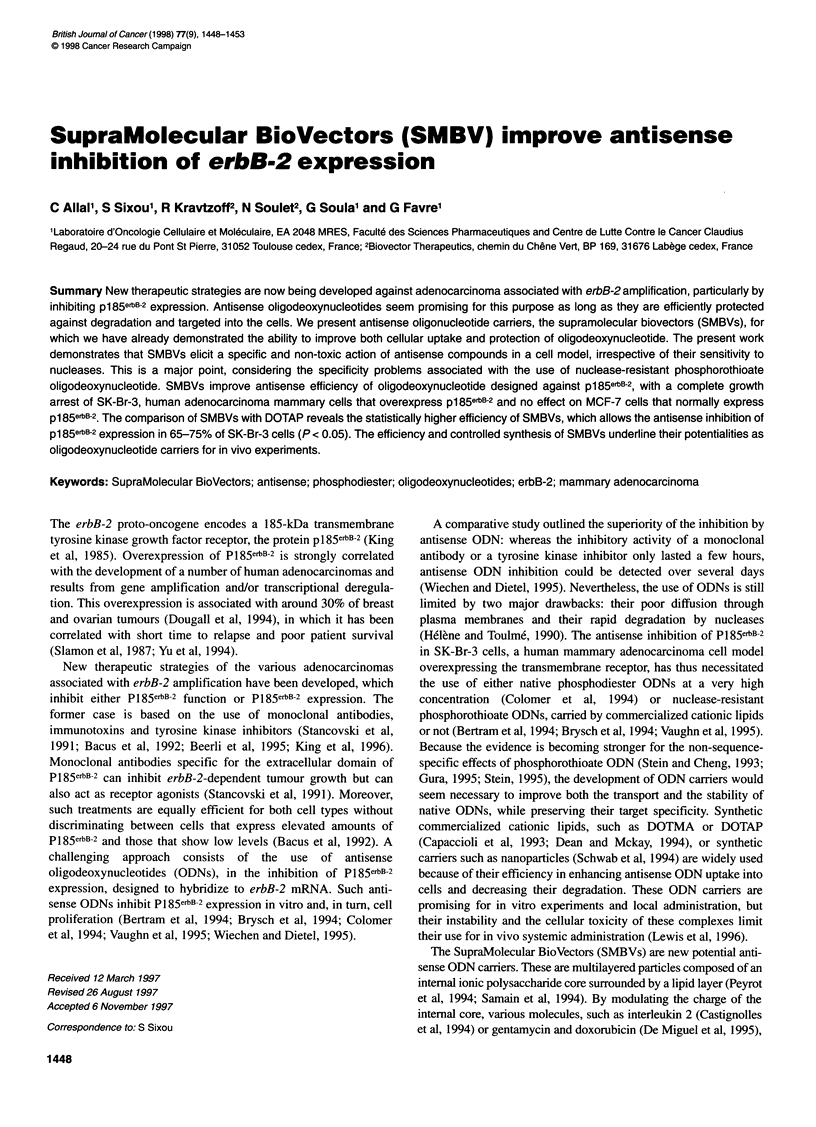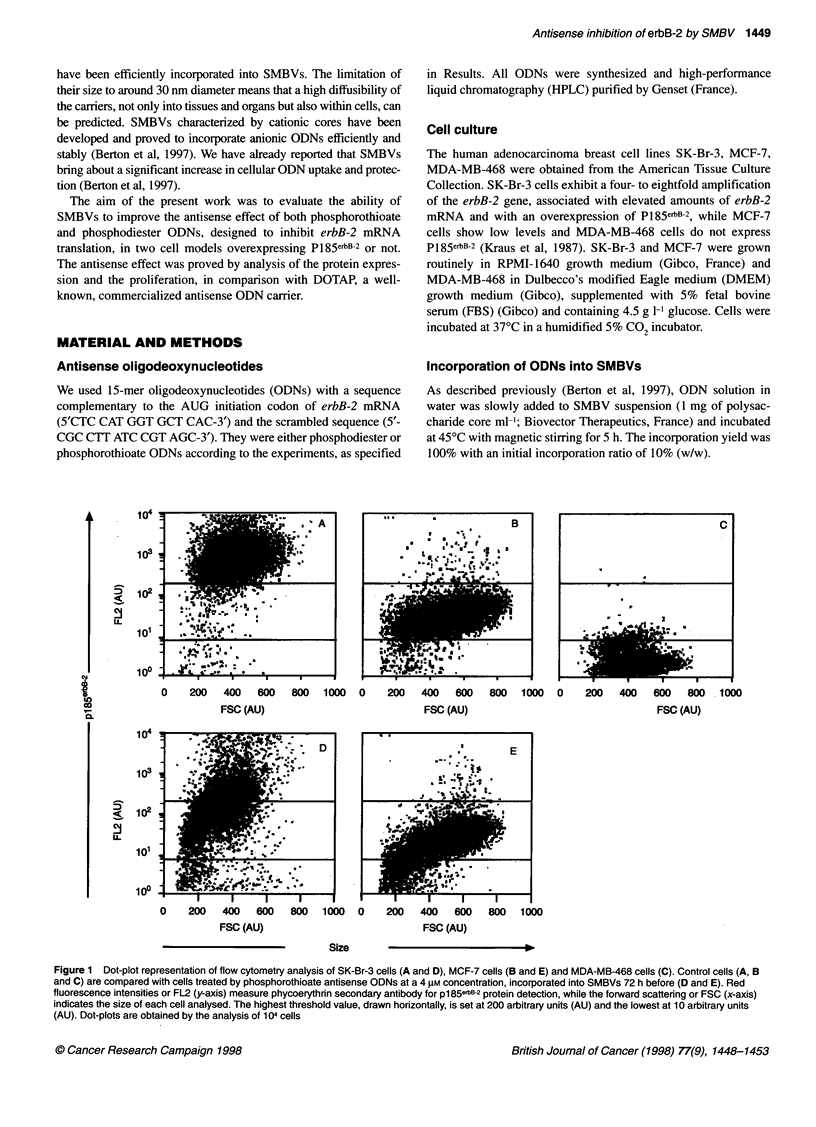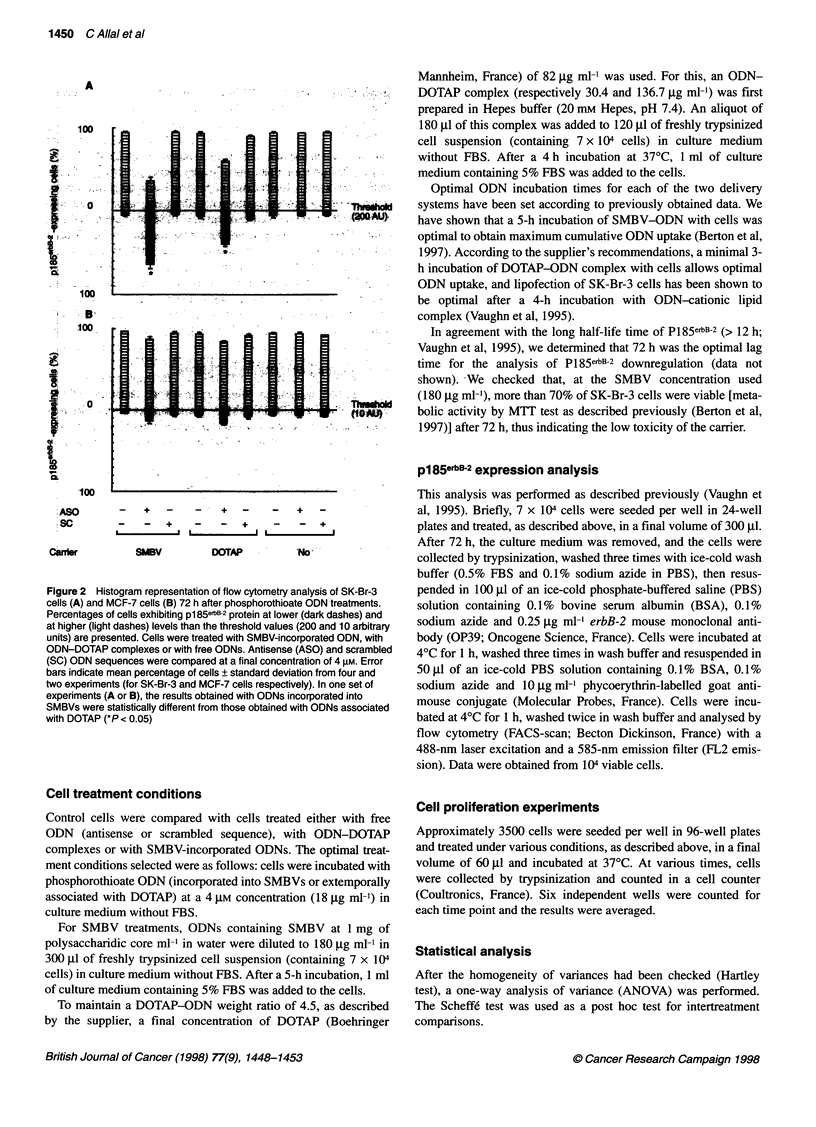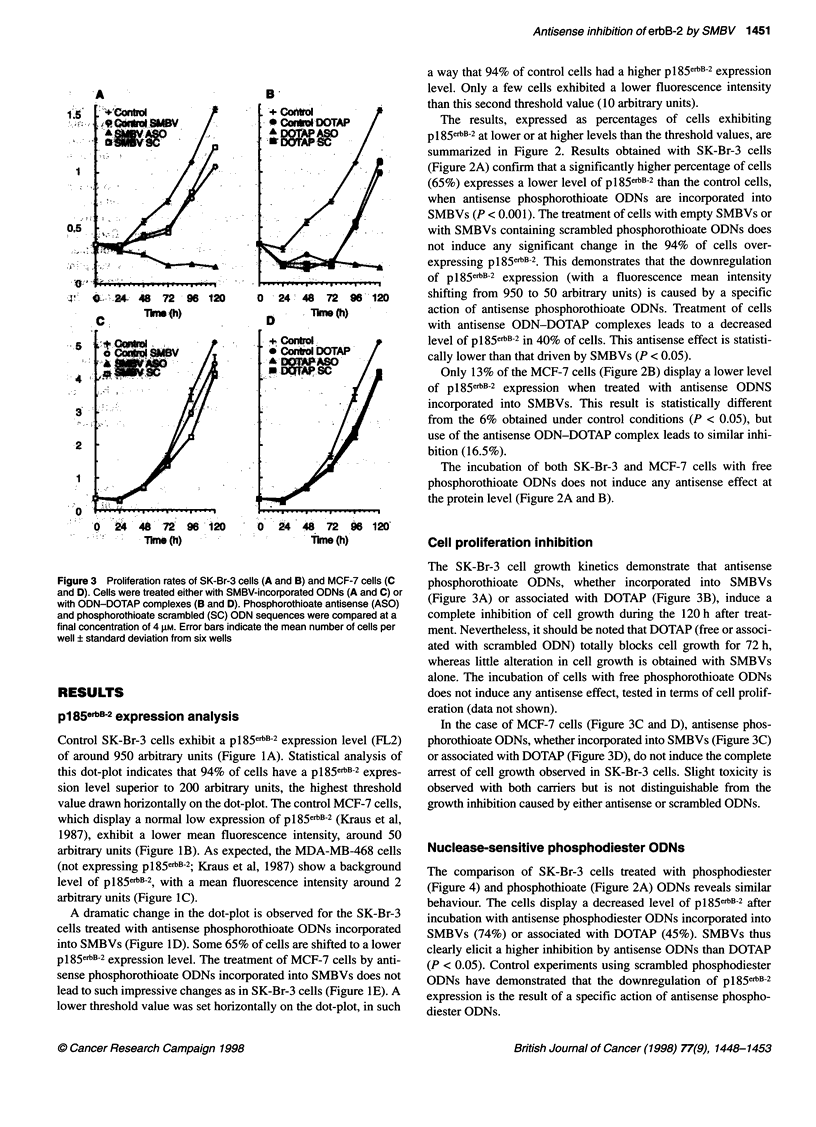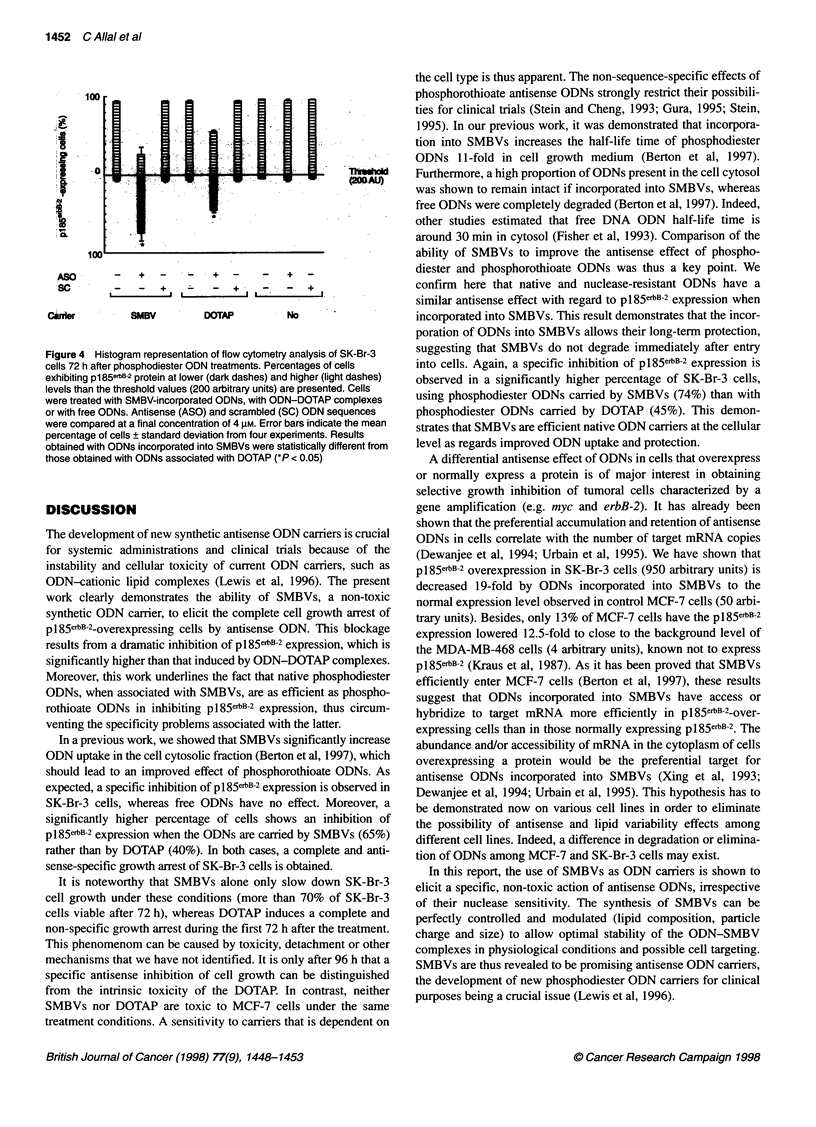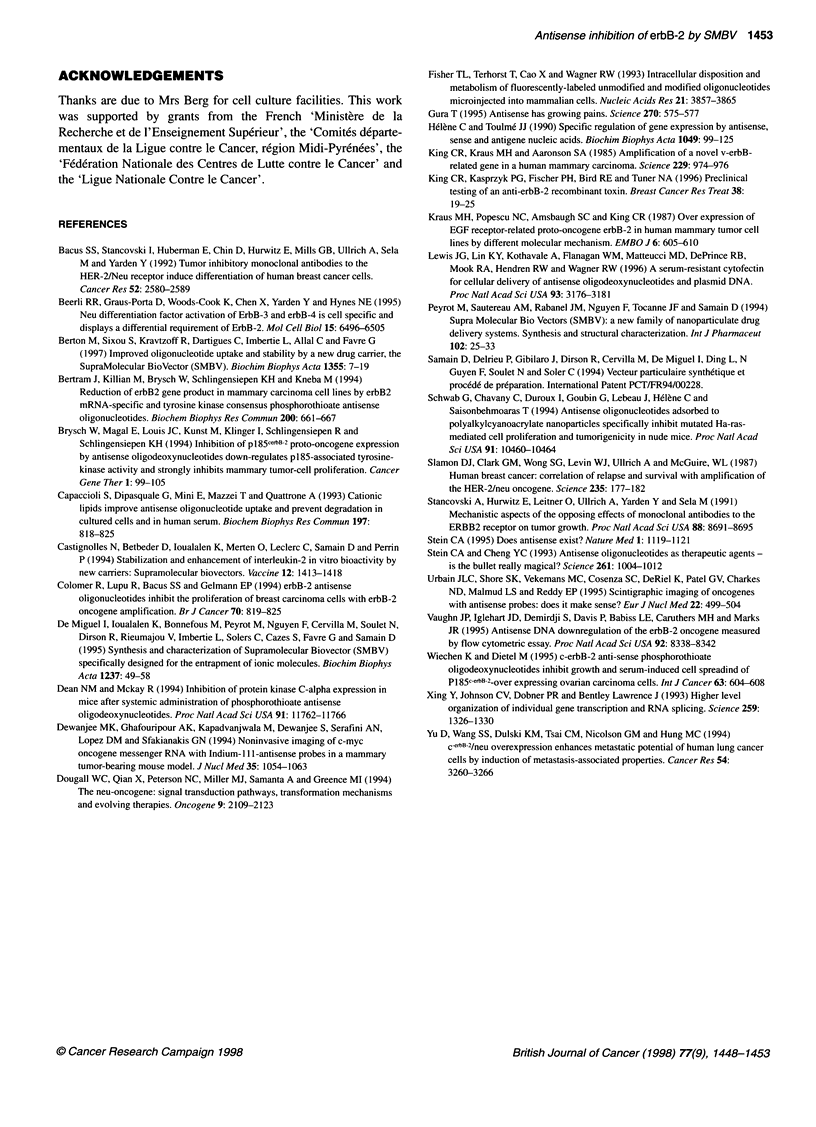# SupraMolecular BioVectors (SMBV) improve antisense inhibition of erbB-2 expression.

**DOI:** 10.1038/bjc.1998.238

**Published:** 1998-05

**Authors:** C. Allal, S. Sixou, R. Kravtzoff, N. Soulet, G. Soula, G. Favre

**Affiliations:** Laboratoire d'Oncologie Cellulaire et MolÃ©culaire, EA 2048 MRES, FacultÃ© des Sciences Pharmaceutiques and Centre de Lutte Contre le Cancer Claudius Regaud, Toulouse, France.

## Abstract

New therapeutic strategies are now being developed against adenocarcinoma associated with erbB-2 amplification, particularly by inhibiting p185erbB-2 expression. Antisense oligodeoxynucleotides seem promising for this purpose as long as they are efficiently protected against degradation and targeted into the cells. We present antisense oligonucleotide carriers, the supramolecular biovectors (SMBVs), for which we have already demonstrated the ability to improve both cellular uptake and protection of oligodeoxynucleotide. The present work demonstrates that SMBVs elicit a specific and non-toxic action of antisense compounds in a cell model, irrespective of their sensitivity to nucleases. This is a major point, considering the specificity problems associated with the use of nuclease-resistant phosphorothioate oligodeoxynucleotide. SMBVs improve antisense efficiency of oligodeoxynucleotide designed against p185erbB-2, with a complete growth arrest of SK-Br-3, human adenocarcinoma mammary cells that overexpress p185erbB-2 and no effect on MCF-7 cells that normally express p185erbB-2. The comparison of SMBVs with DOTAP reveals the statistically higher efficiency of SMBVs, which allows the antisense inhibition of p185erbB-2 expression in 65-75% of SK-Br-3 cells (P < 0.05). The efficiency and controlled synthesis of SMBVs underline their potentialities as oligodeoxynucleotide carriers for in vivo experiments.


British Journal of Cancer (1998) 77(9), 1448–1453
© 1998 Cancer Research Campaign

### SupraMolecular BioVectors (SMBV) improve antisense inhibition of *erbB-2* expression

C Allal[1], S Sixou[1], R Kravtzoff[2], N Soulet[2], G Soula[1] and G Favre[1]

[1]Laboratoire d'Oncologie Cellulaire et Moléculaire, EA 2048 MRES, Faculté des Sciences Pharmaceutiques and Centre de Lutte Contre le Cancer Claudius Regaud, 20–24 rue du Pont St Pierre, 31052 Toulouse cedex, France; [2]Biovector Therapeutics, chemin du Chêne Vert, BP 169, 31676 Labège cedex, France

**Summary** New therapeutic strategies are now being developed against adenocarcinoma associated with *erbB-2* amplification, particularly by inhibiting p185$^{erbB-2}$ expression. Antisense oligodeoxynucleotides seem promising for this purpose as long as they are efficiently protected against degradation and targeted into the cells. We present antisense oligonucleotide carriers, the supramolecular biovectors (SMBVs), for which we have already demonstrated the ability to improve both cellular uptake and protection of oligodeoxynucleotide. The present work demonstrates that SMBVs elicit a specific and non-toxic action of antisense compounds in a cell model, irrespective of their sensitivity to nucleases. This is a major point, considering the specificity problems associated with the use of nuclease-resistant phosphorothioate oligodeoxynucleotide. SMBVs improve antisense efficiency of oligodeoxynucleotide designed against p185$^{erbB-2}$, with a complete growth arrest of SK-Br-3, human adenocarcinoma mammary cells that overexpress p185$^{erbB-2}$ and no effect on MCF-7 cells that normally express p185$^{erbB-2}$. The comparison of SMBVs with DOTAP reveals the statistically higher efficiency of SMBVs, which allows the antisense inhibition of p185$^{erbB-2}$ expression in 65–75% of SK-Br-3 cells ($P < 0.05$). The efficiency and controlled synthesis of SMBVs underline their potentialities as oligodeoxynucleotide carriers for in vivo experiments.

**Keywords:** SupraMolecular BioVectors; antisense; phosphodiester; oligodeoxynucleotides; erbB-2; mammary adenocarcinoma

The *erbB-2* proto-oncogene encodes a 185-kDa transmembrane tyrosine kinase growth factor receptor, the protein p185$^{erbB-2}$ (King et al, 1985). Overexpression of P185$^{erbB-2}$ is strongly correlated with the development of a number of human adenocarcinomas and results from gene amplification and/or transcriptional deregulation. This overexpression is associated with around 30% of breast and ovarian tumours (Dougall et al, 1994), in which it has been correlated with short time to relapse and poor patient survival (Slamon et al, 1987; Yu et al, 1994).

New therapeutic strategies of the various adenocarcinomas associated with *erbB-2* amplification have been developed, which inhibit either P185$^{erbB-2}$ function or P185$^{erbB-2}$ expression. The former case is based on the use of monoclonal antibodies, immunotoxins and tyrosine kinase inhibitors (Stancovski et al, 1991; Bacus et al, 1992; Beerli et al, 1995; King et al, 1996). Monoclonal antibodies specific for the extracellular domain of P185$^{erbB-2}$ can inhibit *erbB-2*-dependent tumour growth but can also act as receptor agonists (Stancovski et al, 1991). Moreover, such treatments are equally efficient for both cell types without discriminating between cells that express elevated amounts of P185$^{erbB-2}$ and those that show low levels (Bacus et al, 1992). A challenging approach consists of the use of antisense oligodeoxynucleotides (ODNs), in the inhibition of P185$^{erbB-2}$ expression, designed to hybridize to *erbB-2* mRNA. Such antisense ODNs inhibit P185$^{erbB-2}$ expression in vitro and, in turn, cell proliferation (Bertram et al, 1994; Brysch et al, 1994; Colomer et al, 1994; Vaughn et al, 1995; Wiechen and Dietel, 1995).

*Received 12 March 1997*
*Revised 26 August 1997*
*Accepted 6 November 1997*

*Correspondence to:* S Sixou

A comparative study outlined the superiority of the inhibition by antisense ODN: whereas the inhibitory activity of a monoclonal antibody or a tyrosine kinase inhibitor only lasted a few hours, antisense ODN inhibition could be detected over several days (Wiechen and Dietel, 1995). Nevertheless, the use of ODNs is still limited by two major drawbacks: their poor diffusion through plasma membranes and their rapid degradation by nucleases (Hélène and Toulmé, 1990). The antisense inhibition of P185$^{erbB-2}$ in SK-Br-3 cells, a human mammary adenocarcinoma cell model overexpressing the transmembrane receptor, has thus necessitated the use of either native phosphodiester ODNs at a very high concentration (Colomer et al, 1994) or nuclease-resistant phosphorothioate ODNs, carried by commercialized cationic lipids or not (Bertram et al, 1994; Brysch et al, 1994; Vaughn et al, 1995). Because the evidence is becoming stronger for the non-sequence-specific effects of phosphorothioate ODN (Stein and Cheng, 1993; Gura, 1995; Stein, 1995), the development of ODN carriers would seem necessary to improve both the transport and the stability of native ODNs, while preserving their target specificity. Synthetic commercialized cationic lipids, such as DOTMA or DOTAP (Capaccioli et al, 1993; Dean and Mckay, 1994), or synthetic carriers such as nanoparticles (Schwab et al, 1994) are widely used because of their efficiency in enhancing antisense ODN uptake into cells and decreasing their degradation. These ODN carriers are promising for in vitro experiments and local administration, but their instability and the cellular toxicity of these complexes limit their use for in vivo systemic administration (Lewis et al, 1996).

The SupraMolecular BioVectors (SMBVs) are new potential antisense ODN carriers. These are multilayered particles composed of an internal ionic polysaccharide core surrounded by a lipid layer (Peyrot et al, 1994; Samain et al, 1994). By modulating the charge of the internal core, various molecules, such as interleukin 2 (Castignolles et al, 1994) or gentamycin and doxorubicin (De Miguel et al, 1995), have been efficiently incorporated into SMBVs. The limitation of their size to around 30 nm diameter means that a high diffusibility of the carriers, not only into tissues and organs but also within cells, can be predicted. SMBVs characterized by cationic cores have been developed and proved to incorporate anionic ODNs efficiently and stably (Berton et al, 1997). We have already reported that SMBVs bring about a significant increase in cellular ODN uptake and protection (Berton et al, 1997).

The aim of the present work was to evaluate the ability of SMBVs to improve the antisense effect of both phosphorothioate and phosphodiester ODNs, designed to inhibit *erbB-2* mRNA translation, in two cell models overexpressing P185$^{erbB-2}$ or not. The antisense effect was proved by analysis of the protein expression and the proliferation, in comparison with DOTAP, a well-known, commercialized antisense ODN carrier.

### MATERIAL AND METHODS

### Antisense oligodeoxynucleotides

We used 15-mer oligodeoxynucleotides (ODNs) with a sequence complementary to the AUG initiation codon of *erbB-2* mRNA (5′CTC CAT GGT GCT CAC-3′) and the scrambled sequence (5′-CGC CTT ATC CGT AGC-3′). They were either phosphodiester or phosphorothioate ODNs according to the experiments, as specified in Results. All ODNs were synthesized and high-performance liquid chromatography (HPLC) purified by Genset (France).

### Cell culture

The human adenocarcinoma breast cell lines SK-Br-3, MCF-7, MDA-MB-468 were obtained from the American Tissue Culture Collection. SK-Br-3 cells exhibit a four- to eightfold amplification of the *erbB-2* gene, associated with elevated amounts of *erbB-2* mRNA and with an overexpression of P185$^{erbB-2}$, while MCF-7 cells show low levels and MDA-MB-468 cells do not express P185$^{erbB-2}$ (Kraus et al, 1987). SK-Br-3 and MCF-7 were grown routinely in RPMI-1640 growth medium (Gibco, France) and MDA-MB-468 in Dulbecco's modified Eagle medium (DMEM) growth medium (Gibco), supplemented with 5% fetal bovine serum (FBS) (Gibco) and containing 4.5 g $l^{-1}$ glucose. Cells were incubated at 37°C in a humidified 5% $CO_2$ incubator.

### Incorporation of ODNs into SMBVs

As described previously (Berton et al, 1997), ODN solution in water was slowly added to SMBV suspension (1 mg of polysaccharide core $ml^{-1}$; Biovector Therapeutics, France) and incubated at 45°C with magnetic stirring for 5 h. The incorporation yield was 100% with an initial incorporation ratio of 10% (w/w).

**Figure 1** Dot-plot representation of flow cytometry analysis of SK-Br-3 cells (**A** and **D**), MCF-7 cells (**B** and **E**) and MDA-MB-468 cells (**C**). Control cells (**A**, **B** and **C**) are compared with cells treated by phosphorothioate antisense ODNs at a 4 μM concentration, incorporated into SMBVs 72 h before (**D** and **E**). Red fluorescence intensities or FL2 (*y*-axis) measure phycoerythrin secondary antibody for p185$^{erbB-2}$ protein detection, while the forward scattering or FSC (*x*-axis) indicates the size of each cell analysed. The highest threshold value, drawn horizontally, is set at 200 arbitrary units (AU) and the lowest at 10 arbitrary units (AU). Dot-plots are obtained by the analysis of $10^4$ cells

© Cancer Research Campaign 1998

**Figure 2** Histogram representation of flow cytometry analysis of SK-Br-3 cells (**A**) and MCF-7 cells (**B**) 72 h after phosphorothioate ODN treatments. Percentages of cells exhibiting p185$^{erbB-2}$ protein at lower (dark dashes) and at higher (light dashes) levels than the threshold values (200 and 10 arbitrary units) are presented. Cells were treated with SMBV-incorporated ODN, with ODN–DOTAP complexes or with free ODNs. Antisense (ASO) and scrambled (SC) ODN sequences were compared at a final concentration of 4 μM. Error bars indicate mean percentage of cells ± standard deviation from four and two experiments (for SK-Br-3 and MCF-7 cells respectively). In one set of experiments (**A** or **B**), the results obtained with ODNs incorporated into SMBVs were statistically different from those obtained with ODNs associated with DOTAP (*$P < 0.05$)

### Cell treatment conditions

Control cells were compared with cells treated either with free ODN (antisense or scrambled sequence), with ODN–DOTAP complexes or with SMBV-incorporated ODNs. The optimal treatment conditions selected were as follows: cells were incubated with phosphorothioate ODN (incorporated into SMBVs or extemporally associated with DOTAP) at a 4 μM concentration (18 μg ml$^{-1}$) in culture medium without FBS.

For SMBV treatments, ODNs containing SMBV at 1 mg of polysaccharidic core ml$^{-1}$ in water were diluted to 180 μg ml$^{-1}$ in 300 μl of freshly trypsinized cell suspension (containing 7 × 10$^4$ cells) in culture medium without FBS. After a 5-h incubation, 1 ml of culture medium containing 5% FBS was added to the cells.

To maintain a DOTAP–ODN weight ratio of 4.5, as described by the supplier, a final concentration of DOTAP (Boehringer Mannheim, France) of 82 μg ml$^{-1}$ was used. For this, an ODN–DOTAP complex (respectively 30.4 and 136.7 μg ml$^{-1}$) was first prepared in Hepes buffer (20 mM Hepes, pH 7.4). An aliquot of 180 μl of this complex was added to 120 μl of freshly trypsinized cell suspension (containing 7 × 10$^4$ cells) in culture medium without FBS. After a 4 h incubation at 37°C, 1 ml of culture medium containing 5% FBS was added to the cells.

Optimal ODN incubation times for each of the two delivery systems have been set according to previously obtained data. We have shown that a 5-h incubation of SMBV–ODN with cells was optimal to obtain maximum cumulative ODN uptake (Berton et al, 1997). According to the supplier's recommendations, a minimal 3-h incubation of DOTAP–ODN complex with cells allows optimal ODN uptake, and lipofection of SK-Br-3 cells has been shown to be optimal after a 4-h incubation with ODN–cationic lipid complex (Vaughn et al, 1995).

In agreement with the long half-life time of P185$^{erbB-2}$ (> 12 h; Vaughn et al, 1995), we determined that 72 h was the optimal lag time for the analysis of P185$^{erbB-2}$ downregulation (data not shown). We checked that, at the SMBV concentration used (180 μg ml$^{-1}$), more than 70% of SK-Br-3 cells were viable [metabolic activity by MTT test as described previously (Berton et al, 1997)] after 72 h, thus indicating the low toxicity of the carrier.

### p185$^{erbB-2}$ expression analysis

This analysis was performed as described previously (Vaughn et al, 1995). Briefly, 7 × 10$^4$ cells were seeded per well in 24-well plates and treated, as described above, in a final volume of 300 μl. After 72 h, the culture medium was removed, and the cells were collected by trypsinization, washed three times with ice-cold wash buffer (0.5% FBS and 0.1% sodium azide in PBS), then resuspended in 100 μl of an ice-cold phosphate-buffered saline (PBS) solution containing 0.1% bovine serum albumin (BSA), 0.1% sodium azide and 0.25 μg ml$^{-1}$ *erbB-2* mouse monoclonal antibody (OP39; Oncogene Science, France). Cells were incubated at 4°C for 1 h, washed three times in wash buffer and resuspended in 50 μl of an ice-cold PBS solution containing 0.1% BSA, 0.1% sodium azide and 10 μg ml$^{-1}$ phycoerythrin-labelled goat anti-mouse conjugate (Molecular Probes, France). Cells were incubated at 4°C for 1 h, washed twice in wash buffer and analysed by flow cytometry (FACS-scan; Becton Dickinson, France) with a 488-nm laser excitation and a 585-nm emission filter (FL2 emission). Data were obtained from 10$^4$ viable cells.

### Cell proliferation experiments

Approximately 3500 cells were seeded per well in 96-well plates and treated under various conditions, as described above, in a final volume of 60 μl and incubated at 37°C. At various times, cells were collected by trypsinization and counted in a cell counter (Coultronics, France). Six independent wells were counted for each time point and the results were averaged.

### Statistical analysis

After the homogeneity of variances had been checked (Hartley test), a one-way analysis of variance (ANOVA) was performed. The Scheffé test was used as a post hoc test for intertreatment comparisons.

*© Cancer Research Campaign 1998*

**Figure 3** Proliferation rates of SK-Br-3 cells (**A** and **B**) and MCF-7 cells (**C** and **D**). Cells were treated either with SMBV-incorporated ODNs (**A** and **C**) or with ODN–DOTAP complexes (**B** and **D**). Phosphorothioate antisense (ASO) and phosphorothioate scrambled (SC) ODN sequences were compared at a final concentration of 4 μM. Error bars indicate the mean number of cells per well ± standard deviation from six wells

### RESULTS

### p185$^{erbB-2}$ expression analysis

Control SK-Br-3 cells exhibit a p185$^{erbB-2}$ expression level (FL2) of around 950 arbitrary units (Figure 1A). Statistical analysis of this dot-plot indicates that 94% of cells have a p185$^{erbB-2}$ expression level superior to 200 arbitrary units, the highest threshold value drawn horizontally on the dot-plot. The control MCF-7 cells, which display a normal low expression of p185$^{erbB-2}$ (Kraus et al, 1987), exhibit a lower mean fluorescence intensity, around 50 arbitrary units (Figure 1B). As expected, the MDA-MB-468 cells (not expressing p185$^{erbB-2}$; Kraus et al, 1987) show a background level of p185$^{erbB-2}$, with a mean fluorescence intensity around 2 arbitrary units (Figure 1C).

A dramatic change in the dot-plot is observed for the SK-Br-3 cells treated with antisense phosphorothioate ODNs incorporated into SMBVs (Figure 1D). Some 65% of cells are shifted to a lower p185$^{erbB-2}$ expression level. The treatment of MCF-7 cells by antisense phosphorothioate ODNs incorporated into SMBVs does not lead to such impressive changes as in SK-Br-3 cells (Figure 1E). A lower threshold value was set horizontally on the dot-plot, in such a way that 94% of control cells had a higher p185$^{erbB-2}$ expression level. Only a few cells exhibited a lower fluorescence intensity than this second threshold value (10 arbitrary units).

The results, expressed as percentages of cells exhibiting p185$^{erbB-2}$ at lower or at higher levels than the threshold values, are summarized in Figure 2. Results obtained with SK-Br-3 cells (Figure 2A) confirm that a significantly higher percentage of cells (65%) expresses a lower level of p185$^{erbB-2}$ than the control cells, when antisense phosphorothioate ODNs are incorporated into SMBVs ($P < 0.001$). The treatment of cells with empty SMBVs or with SMBVs containing scrambled phosphorothioate ODNs does not induce any significant change in the 94% of cells over-expressing p185$^{erbB-2}$. This demonstrates that the downregulation of p185$^{erbB-2}$ expression (with a fluorescence mean intensity shifting from 950 to 50 arbitrary units) is caused by a specific action of antisense phosphorothioate ODNs. Treatment of cells with antisense ODN–DOTAP complexes leads to a decreased level of p185$^{erbB-2}$ in 40% of cells. This antisense effect is statistically lower than that driven by SMBVs ($P < 0.05$).

Only 13% of the MCF-7 cells (Figure 2B) display a lower level of p185$^{erbB-2}$ expression when treated with antisense ODNS incorporated into SMBVs. This result is statistically different from the 6% obtained under control conditions ($P < 0.05$), but use of the antisense ODN–DOTAP complex leads to similar inhibition (16.5%).

The incubation of both SK-Br-3 and MCF-7 cells with free phosphorothioate ODNs does not induce any antisense effect at the protein level (Figure 2A and B).

### Cell proliferation inhibition

The SK-Br-3 cell growth kinetics demonstrate that antisense phosphorothioate ODNs, whether incorporated into SMBVs (Figure 3A) or associated with DOTAP (Figure 3B), induce a complete inhibition of cell growth during the 120 h after treatment. Nevertheless, it should be noted that DOTAP (free or associated with scrambled ODN) totally blocks cell growth for 72 h, whereas little alteration in cell growth is obtained with SMBVs alone. The incubation of cells with free phosphorothioate ODNs does not induce any antisense effect, tested in terms of cell proliferation (data not shown).

In the case of MCF-7 cells (Figure 3C and D), antisense phosphorothioate ODNs, whether incorporated into SMBVs (Figure 3C) or associated with DOTAP (Figure 3D), do not induce the complete arrest of cell growth observed in SK-Br-3 cells. Slight toxicity is observed with both carriers but is not distinguishable from the growth inhibition caused by either antisense or scrambled ODNs.

### Nuclease-sensitive phosphodiester ODNs

The comparison of SK-Br-3 cells treated with phosphodiester (Figure 4) and phosphothioate (Figure 2A) ODNs reveals similar behaviour. The cells display a decreased level of p185$^{erbB-2}$ after incubation with antisense phosphodiester ODNs incorporated into SMBVs (74%) or associated with DOTAP (45%). SMBVs thus clearly elicit a higher inhibition by antisense ODNs than DOTAP ($P < 0.05$). Control experiments using scrambled phosphodiester ODNs have demonstrated that the downregulation of p185$^{erbB-2}$ expression is the result of a specific action of antisense phosphodiester ODNs.

© Cancer Research Campaign 1998

**Figure 4** Histogram representation of flow cytometry analysis of SK-Br-3 cells 72 h after phosphodiester ODN treatments. Percentages of cells exhibiting p185$^{erbB-2}$ protein at lower (dark dashes) and higher (light dashes) levels than the threshold values (200 arbitrary units) are presented. Cells were treated with SMBV-incorporated ODNs, with ODN–DOTAP complexes or with free ODNs. Antisense (ASO) and scrambled (SC) ODN sequences were compared at a final concentration of 4 μM. Error bars indicate the mean percentage of cells ± standard deviation from four experiments. Results obtained with ODNs incorporated into SMBVs were statistically different from those obtained with ODNs associated with DOTAP (*$P < 0.05$)

### DISCUSSION

The development of new synthetic antisense ODN carriers is crucial for systemic administrations and clinical trials because of the instability and cellular toxicity of current ODN carriers, such as ODN–cationic lipid complexes (Lewis et al, 1996). The present work clearly demonstrates the ability of SMBVs, a non-toxic synthetic ODN carrier, to elicit the complete cell growth arrest of p185$^{erbB-2}$-overexpressing cells by antisense ODN. This blockage results from a dramatic inhibition of p185$^{erbB-2}$ expression, which is significantly higher than that induced by ODN–DOTAP complexes. Moreover, this work underlines the fact that native phosphodiester ODNs, when associated with SMBVs, are as efficient as phosphorothioate ODNs in inhibiting p185$^{erbB-2}$ expression, thus circumventing the specificity problems associated with the latter.

In a previous work, we showed that SMBVs significantly increase ODN uptake in the cell cytosolic fraction (Berton et al, 1997), which should lead to an improved effect of phosphorothioate ODNs. As expected, a specific inhibition of p185$^{erbB-2}$ expression is observed in SK-Br-3 cells, whereas free ODNs have no effect. Moreover, a significantly higher percentage of cells shows an inhibition of p185$^{erbB-2}$ expression when the ODNs are carried by SMBVs (65%) rather than by DOTAP (40%). In both cases, a complete and antisense-specific growth arrest of SK-Br-3 cells is obtained.

It is noteworthy that SMBVs alone only slow down SK-Br-3 cell growth under these conditions (more than 70% of SK-Br-3 cells viable after 72 h), whereas DOTAP induces a complete and non-specific growth arrest during the first 72 h after the treatment. This phenomenom can be caused by toxicity, detachment or other mechanisms that we have not identified. It is only after 96 h that a specific antisense inhibition of cell growth can be distinguished from the intrinsic toxicity of the DOTAP. In contrast, neither SMBVs nor DOTAP are toxic to MCF-7 cells under the same treatment conditions. A sensitivity to carriers that is dependent on the cell type is thus apparent. The non-sequence-specific effects of phosphorothioate antisense ODNs strongly restrict their possibilities for clinical trials (Stein and Cheng, 1993; Gura, 1995; Stein, 1995). In our previous work, it was demonstrated that incorporation into SMBVs increases the half-life time of phosphodiester ODNs 11-fold in cell growth medium (Berton et al, 1997). Furthermore, a high proportion of ODNs present in the cell cytosol was shown to remain intact if incorporated into SMBVs, whereas free ODNs were completely degraded (Berton et al, 1997). Indeed, other studies estimated that free DNA ODN half-life time is around 30 min in cytosol (Fisher et al, 1993). Comparison of the ability of SMBVs to improve the antisense effect of phosphodiester and phosphorothioate ODNs was thus a key point. We confirm here that native and nuclease-resistant ODNs have a similar antisense effect with regard to p185$^{erbB-2}$ expression when incorporated into SMBVs. This result demonstrates that the incorporation of ODNs into SMBVs allows their long-term protection, suggesting that SMBVs do not degrade immediately after entry into cells. Again, a specific inhibition of p185$^{erbB-2}$ expression is observed in a significantly higher percentage of SK-Br-3 cells, using phosphodiester ODNs carried by SMBVs (74%) than with phosphodiester ODNs carried by DOTAP (45%). This demonstrates that SMBVs are efficient native ODN carriers at the cellular level as regards improved ODN uptake and protection.

A differential antisense effect of ODNs in cells that overexpress or normally express a protein is of major interest in obtaining selective growth inhibition of tumoral cells characterized by a gene amplification (e.g. *myc* and *erbB-2*). It has already been shown that the preferential accumulation and retention of antisense ODNs in cells correlate with the number of target mRNA copies (Dewanjee et al, 1994; Urbain et al, 1995). We have shown that p185$^{erbB-2}$ overexpression in SK-Br-3 cells (950 arbitrary units) is decreased 19-fold by ODNs incorporated into SMBVs to the normal expression level observed in control MCF-7 cells (50 arbitrary units). Besides, only 13% of MCF-7 cells have the p185$^{erbB-2}$ expression lowered 12.5-fold to close to the background level of the MDA-MB-468 cells (4 arbitrary units), known not to express p185$^{erbB-2}$ (Kraus et al, 1987). As it has been proved that SMBVs efficiently enter MCF-7 cells (Berton et al, 1997), these results suggest that ODNs incorporated into SMBVs have access or hybridize to target mRNA more efficiently in p185$^{erbB-2}$-overexpressing cells than in those normally expressing p185$^{erbB-2}$. The abundance and/or accessibility of mRNA in the cytoplasm of cells overexpressing a protein would be the preferential target for antisense ODNs incorporated into SMBVs (Xing et al, 1993; Dewanjee et al, 1994; Urbain et al, 1995). This hypothesis has to be demonstrated now on various cell lines in order to eliminate the possibility of antisense and lipid variability effects among different cell lines. Indeed, a difference in degradation or elimination of ODNs among MCF-7 and SK-Br-3 cells may exist.

In this report, the use of SMBVs as ODN carriers is shown to elicit a specific, non-toxic action of antisense ODNs, irrespective of their nuclease sensitivity. The synthesis of SMBVs can be perfectly controlled and modulated (lipid composition, particle charge and size) to allow optimal stability of the ODN–SMBV complexes in physiological conditions and possible cell targeting. SMBVs are thus revealed to be promising antisense ODN carriers, the development of new phosphodiester ODN carriers for clinical purposes being a crucial issue (Lewis et al, 1996).

© Cancer Research Campaign 1998

### ACKNOWLEDGEMENTS

Thanks are due to Mrs Berg for cell culture facilities. This work was supported by grants from the French 'Ministère de la Recherche et de l'Enseignement Supérieur', the 'Comités départementaux de la Ligue contre le Cancer, région Midi-Pyrénées', the 'Fédération Nationale des Centres de Lutte contre le Cancer' and the 'Ligue Nationale Contre le Cancer'.

### REFERENCES

Bacus SS, Stancovski I, Huberman E, Chin D, Hurwitz E, Mills GB, Ullrich A, Sela M and Yarden Y (1992) Tumor inhibitory monoclonal antibodies to the HER-2/Neu receptor induce differentiation of human breast cancer cells. *Cancer Res* **52**: 2580–2589

Beerli RR, Graus-Porta D, Woods-Cook K, Chen X, Yarden Y and Hynes NE (1995) Neu differentiation factor activation of ErbB-3 and erbB-4 is cell specific and displays a differential requirement of ErbB-2. *Mol Cell Biol* **15**: 6496–6505

Berton M, Sixou S, Kravtzoff R, Dartigues C, Imbertie L, Allal C and Favre G (1997) Improved oligonucleotide uptake and stability by a new drug carrier, the SupraMolecular BioVector (SMBV). *Biochim Biophys Acta* **1355**: 7–19

Bertram J, Killian M, Brysch W, Schlingensiepen KH and Kneba M (1994) Reduction of erbB2 gene product in mammary carcinoma cell lines by erbB2 mRNA-specific and tyrosine kinase consensus phosphorothioate antisense oligonucleotides. *Biochem Biophys Res Commun* **200**: 661–667

Brysch W, Magal E, Louis JC, Kunst M, Klinger I, Schlingensiepen R and Schlingensiepen KH (1994) Inhibition of p185$^{cerbB-2}$ proto-oncogene expression by antisense oligodeoxynucleotides down-regulates p185-associated tyrosine-kinase activity and strongly inhibits mammary tumor-cell proliferation. *Cancer Gene Ther* **1**: 99–105

Capaccioli S, Dipasquale G, Mini E, Mazzei T and Quattrone A (1993) Cationic lipids improve antisense oligonucleotide uptake and prevent degradation in cultured cells and in human serum. *Biochem Biophys Res Commun* **197**: 818–825

Castignolles N, Betbeder D, Ioualalen K, Merten O, Leclerc C, Samain D and Perrin P (1994) Stabilization and enhancement of interleukin-2 in vitro bioactivity by new carriers: Supramolecular biovectors. *Vaccine* **12**: 1413–1418

Colomer R, Lupu R, Bacus SS and Gelmann EP (1994) erbB-2 antisense oligonucleotides inhibit the proliferation of breast carcinoma cells with erbB-2 oncogene amplification. *Br J Cancer* **70**: 819–825

De Miguel I, Ioualalen K, Bonnefous M, Peyrot M, Nguyen F, Cervilla M, Soulet N, Dirson R, Rieumajou V, Imbertie L, Solers C, Cazes S, Favre G and Samain D (1995) Synthesis and characterization of Supramolecular Biovector (SMBV) specifically designed for the entrapment of ionic molecules. *Biochim Biophys Acta* **1237**: 49–58

Dean NM and Mckay R (1994) Inhibition of protein kinase C-alpha expression in mice after systemic administration of phosphorothioate antisense oligodeoxynucleotides. *Proc Natl Acad Sci USA* **91**: 11762–11766

Dewanjee MK, Ghafouripour AK, Kapadvanjwala M, Dewanjee S, Serafini AN, Lopez DM and Sfakianakis GN (1994) Noninvasive imaging of c-myc oncogene messenger RNA with Indium-111-antisense probes in a mammary tumor-bearing mouse model. *J Nucl Med* **35**: 1054–1063

Dougall WC, Qian X, Peterson NC, Miller MJ, Samanta A and Greence MI (1994) The neu-oncogene: signal transduction pathways, transformation mechanisms and evolving therapies. *Oncogene* **9**: 2109–2123

Fisher TL, Terhorst T, Cao X and Wagner RW (1993) Intracellular disposition and metabolism of fluorescently-labeled unmodified and modified oligonucleotides microinjected into mammalian cells. *Nucleic Acids Res* **21**: 3857–3865

Gura T (1995) Antisense has growing pains. *Science* **270**: 575–577

Hélène C and Toulmé JJ (1990) Specific regulation of gene expression by antisense, sense and antigene nucleic acids. *Biochim Biophys Acta* **1049**: 99–125

King CR, Kraus MH and Aaronson SA (1985) Amplification of a novel v-erbB-related gene in a human mammary carcinoma. *Science* **229**: 974–976

King CR, Kasprzyk PG, Fischer PH, Bird RE and Tuner NA (1996) Preclinical testing of an anti-erbB-2 recombinant toxin. *Breast Cancer Res Treat* **38**: 19–25

Kraus MH, Popescu NC, Amsbaugh SC and King CR (1987) Over expression of EGF receptor-related proto-oncogene erbB-2 in human mammary tumor cell lines by different molecular mechanism. *EMBO J* **6**: 605–610

Lewis JG, Lin KY, Kothavale A, Flanagan WM, Matteucci MD, DePrince RB, Mook RA, Hendren RW and Wagner RW (1996) A serum-resistant cytofectin for cellular delivery of antisense oligodeoxynucleotides and plasmid DNA. *Proc Natl Acad Sci USA* **93**: 3176–3181

Peyrot M, Sautereau AM, Rabanel JM, Nguyen F, Tocanne JF and Samain D (1994) Supra Molecular Bio Vectors (SMBV): a new family of nanoparticulate drug delivery systems. Synthesis and structural characterization. *Int J Pharmaceut* **102**: 25–33

Samain D, Delrieu P, Gibilaro J, Dirson R, Cervilla M, De Miguel I, Ding L, N Guyen F, Soulet N and Soler C (1994) Vecteur particulaire synthétique et procédé de préparation. International Patent PCT/FR94/00228.

Schwab G, Chavany C, Duroux I, Goubin G, Lebeau J, Hélène C and Saisonbehmoaras T (1994) Antisense oligonucleotides adsorbed to polyalkylcyanoacrylate nanoparticles specifically inhibit mutated Ha-ras-mediated cell proliferation and tumorigenicity in nude mice. *Proc Natl Acad Sci USA* **91**: 10460–10464

Slamon DJ, Clark GM, Wong SG, Levin WJ, Ullrich A and McGuire, WL (1987) Human breast cancer: correlation of relapse and survival with amplification of the HER-2/neu oncogene. *Science* **235**: 177–182

Stancovski A, Hurwitz E, Leitner O, Ullrich A, Yarden Y and Sela M (1991) Mechanistic aspects of the opposing effects of monoclonal antibodies to the ERBB2 receptor on tumor growth. *Proc Natl Acad Sci USA* **88**: 8691–8695

Stein CA (1995) Does antisense exist? *Nature Med* **1**: 1119–1121

Stein CA and Cheng YC (1993) Antisense oligonucleotides as therapeutic agents – is the bullet really magical? *Science* **261**: 1004–1012

Urbain JLC, Shore SK, Vekemans MC, Cosenza SC, DeRiel K, Patel GV, Charkes ND, Malmud LS and Reddy EP (1995) Scintigraphic imaging of oncogenes with antisense probes: does it make sense? *Eur J Nucl Med* **22**: 499–504

Vaughn JP, Iglehart JD, Demirdji S, Davis P, Babiss LE, Caruthers MH and Marks JR (1995) Antisense DNA downregulation of the erbB-2 oncogene measured by flow cytometric essay. *Proc Natl Acad Sci USA* **92**: 8338–8342

Wiechen K and Dietel M (1995) c-erbB-2 anti-sense phosphorothioate oligodeoxynucleotides inhibit growth and serum-induced cell spreadind of P185$^{c-erbB-2}$-over expressing ovarian carcinoma cells. *Int J Cancer* **63**: 604–608

Xing Y, Johnson CV, Dobner PR and Bentley Lawrence J (1993) Higher level organization of individual gene transcription and RNA splicing. *Science* **259**: 1326–1330

Yu D, Wang SS, Dulski KM, Tsai CM, Nicolson GM and Hung MC (1994) c$^{-erbB-2}$/neu overexpression enhances metastatic potential of human lung cancer cells by induction of metastasis-associated properties. *Cancer Res* **54**: 3260–3266

© Cancer Research Campaign 1998